# Rib-fistula in a Cow

**Published:** 1896-04

**Authors:** J. H. Cramer


RIB-FISTULA IN A COW, by J. H. Cramer. Some time ago I was summoned by a farmer in Ambt-Almelo to look at his four-year-old black-brindled cow, which had a diffuse swelling about the size of a child's head on the right rib-wall near the seventh and eighth ribs, just above the elbow. In the middle of the swelling mentioned there was a very small opening which had been discharging for two weeks a small quantity of offensive pus, while the patient, especially in the last four days, had fallen off in her feeding and in the secretion of milk. Pushing down with the probe into the opening of the fistula, I found that it was impossible to penetrate further than a few centimetres, so that I was compelled to make a good-sized opening with the bistoury.

After the tissue of the swelling had been partially destroyed I succeeded in pressing the finger deeper down. I met very quickly a sharp object, so that I supposed I had to deal with a darning-needle or some other pointed object. After some manipulation, however, it became clear that there was a necrotic piece of bone with a sharp point at the bottom. I succeeded with some difficulty in separating the piece of dead bone mentioned from the ribs, and in removing it it seemed to be about 7 cm. long and half as broad. The usual treatment of open wounds produced a perfect healing after some weeks.

The owner of the cow informed me, in conclusion, that the patient about five weeks before had been hooked by another cow in the pasture, in consequence of which a swelling about the size of one's fist had arisen. I think I am justified in supposing that this injury produced ultimately the sequestration of dead bone.—*Tijdschr. voor Veeartsenijkunde*, January number.

